# Supplementary figures and images for: Distinct function of SPL genes in age-related resistance in Arabidopsis
Source: PLoS Pathog. 2023 Mar 22;19(3):e1011218. doi: 10.1371/journal.ppat.1011218 (PMC10069772; doi:10.1371/journal.ppat.1011218)

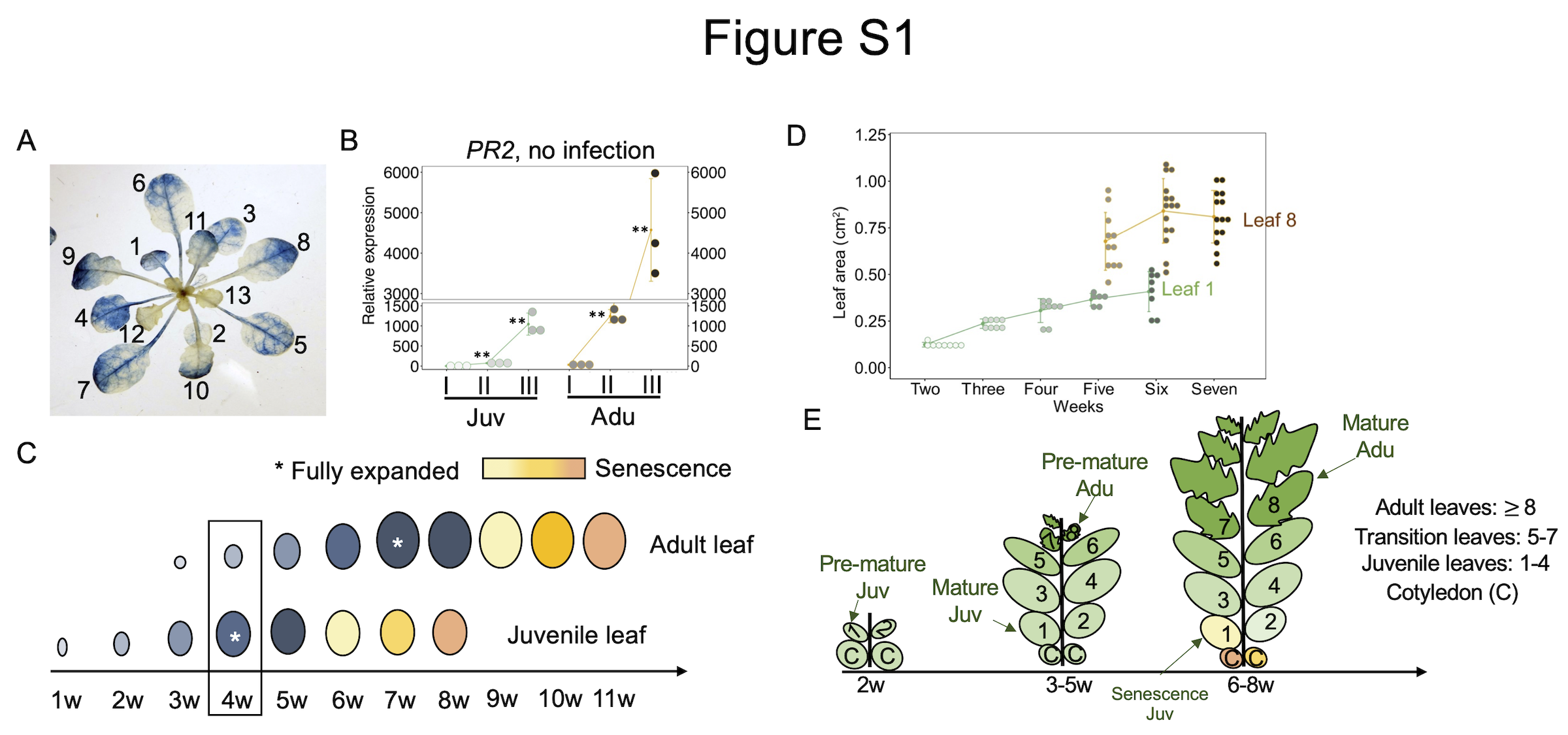

Supplement: S1 Fig — A, ontogenic-associated promoter activities of proPR2::GUS in an uninfected plant. Note the high activity in fully expanded juvenile leaves (1–7) and low in young adult leaves (12–13). The indicated leaf numbers were based on the order of the leaves on shoot. B, the incremental expression of PR2 gene spanning the expansion of juvenile and adult leaves. I, premature leaves. II, intermediate premature leaves. III, mature leaves. C, an outline of the ontogenic maturation of a juvenile leaf and an adult leaf. The boxed region indicates distinct ontogenic age of juvenile and adult leaves from the same plant; asteroids indicate juvenile and adult leaves of the same ontogenic age. D, the quantified leaf expansion rate in juvenile and adult leaves. Leaf areas were quantified and normalized through Fiji software. E, a cartoon depiction of shoot development and leaf maturation that are concurrent during the vegetative phase change. (TIFF) [file ppat.1011218.s001.tiff]

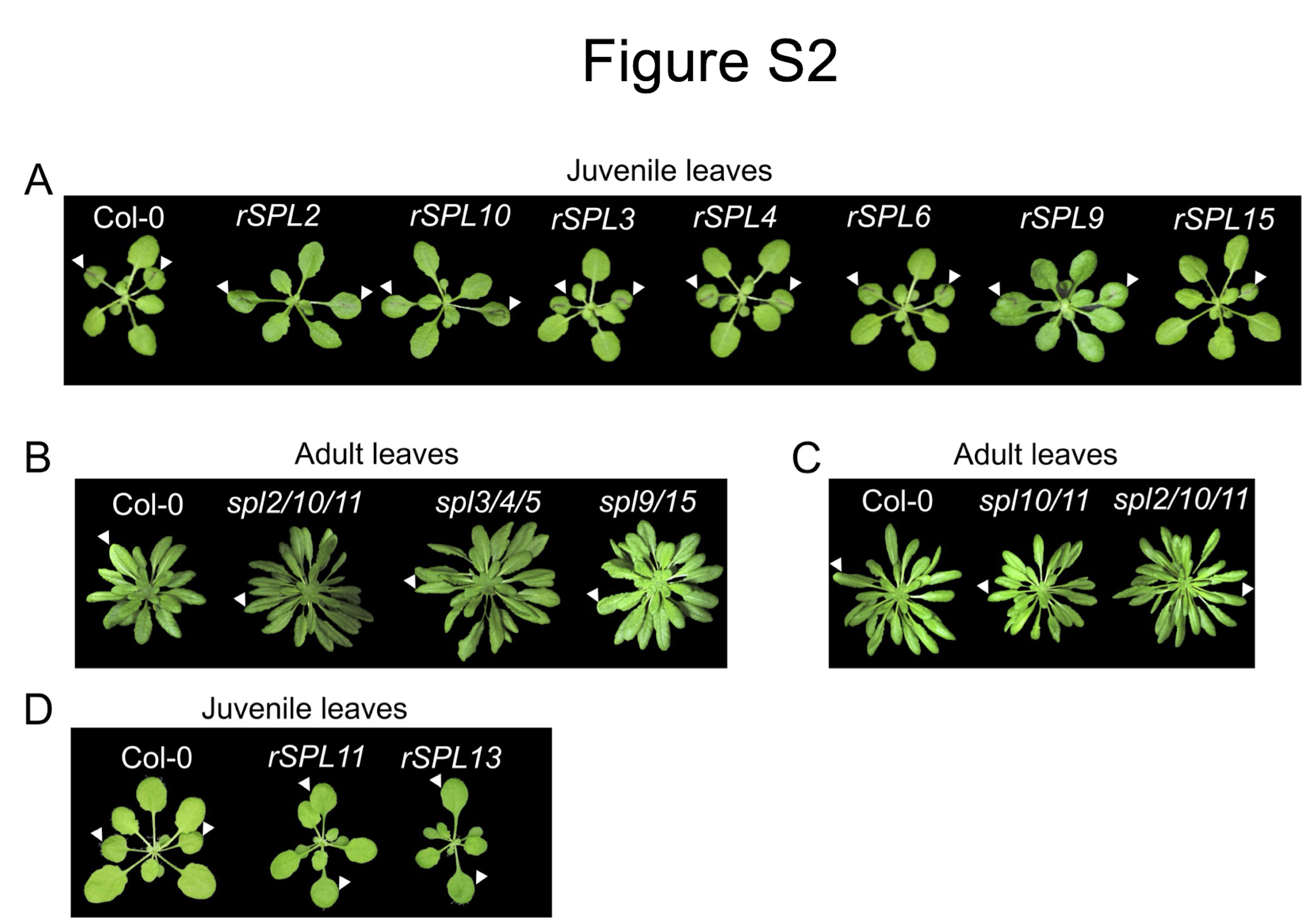

Supplement: S2 Fig — A and D, arrows indicate leaf 1–2 of juvenile Col-0 and rSPLs. B-C, arrows indicate representative adult leaves of Col-0 and spl mutants. (TIFF) [file ppat.1011218.s002.tiff]

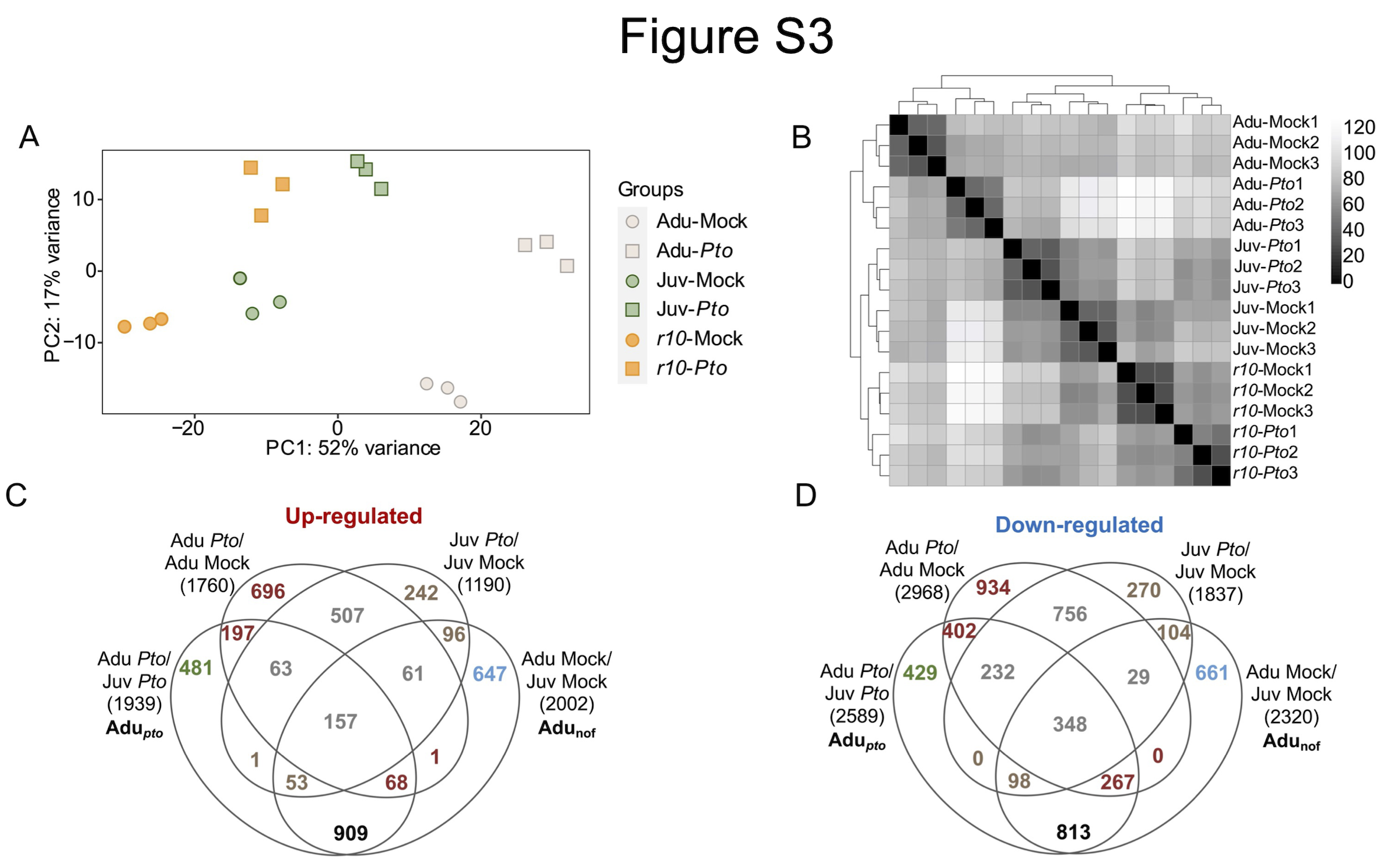

Supplement: S3 Fig — A, principal component analysis showed that the effect of age and genotype likely explained 52% variance of the samples, and Pto infection effect likely explained 17% variance of the samples. B, the sample-to-sample distance matrix showed that biological replicates (Mock1-3 and Pto1-3) were well correlated (in close distance) within per genotype per treatment. The column names of the matrix are in the same order as the row names—from the “Adu-Mock1” (the first on the left) to the “r10-Pto3” (the first on the right). C, venn diagrams of Adu-DEGs generated from the indicated pair-wise comparisons. Color-coding of numbers, adult-specifically Pto-triggered DEGs (red), Juvenile-specifically triggered (brown), commonly triggered in both adults and juveniles, i.e., shared (grey), and overlap DEGs between Adunof and Adupto (black). Green numbers indicate the 20.1% synergistic DEGs that mentioned in the main text. Blue numbers refer to DEGs that were Adunof but not Adupto. Adult preferentially Pto-triggered DEGs are listed in S3 Table. (TIFF) [file ppat.1011218.s003.tiff]

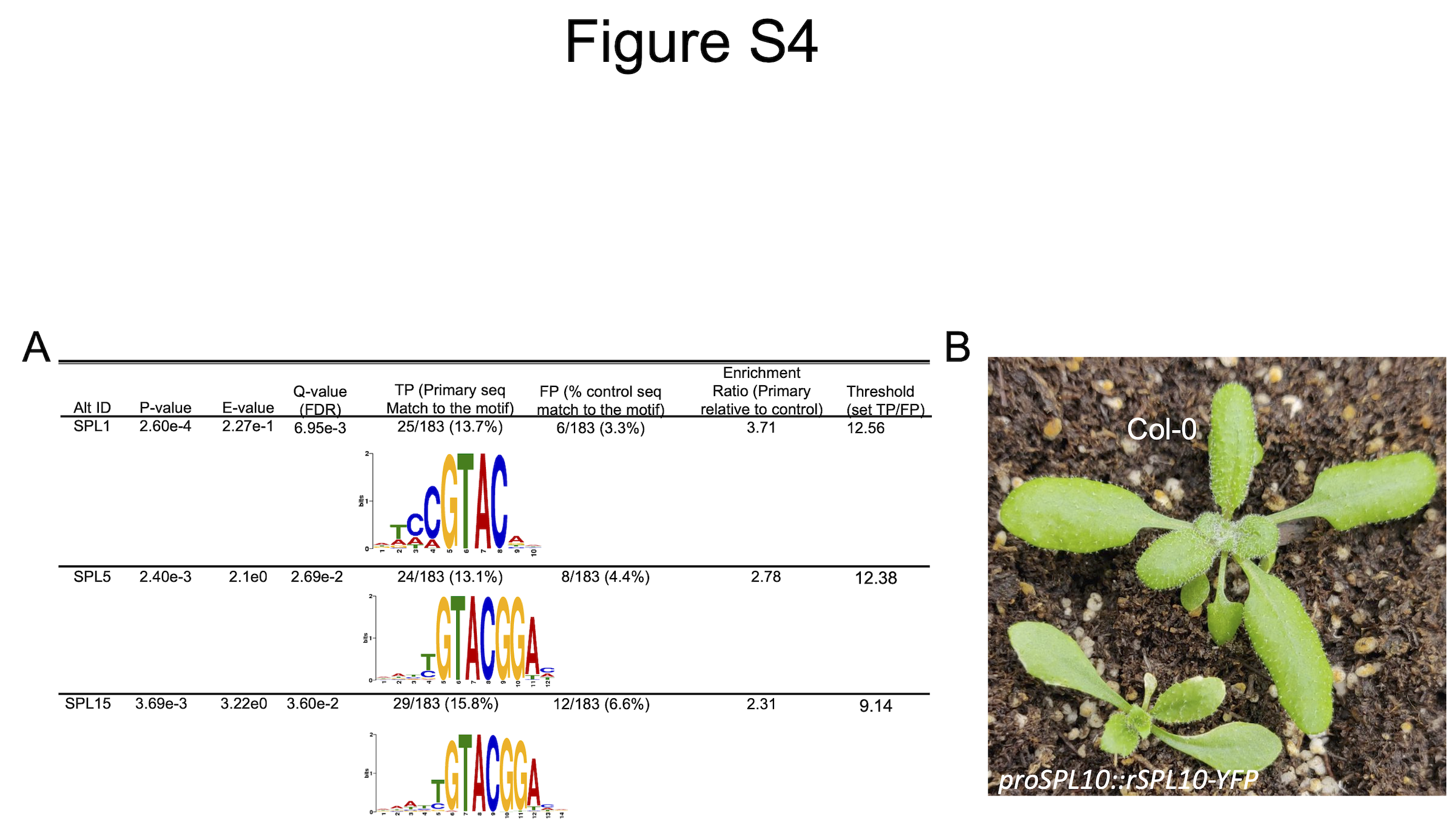

Supplement: S4 Fig — A. Frequency-based DNA logos are shown for each enriched motif. The analysis was performed in the simple enrichment analysis-MEME Suite. Details were described in the method section. B, plant phenotype of proSPL10::rSPL10-YFP. Note the elongated leaves 1 and 2 in the transgenic plant. (TIFF) [file ppat.1011218.s004.tiff]

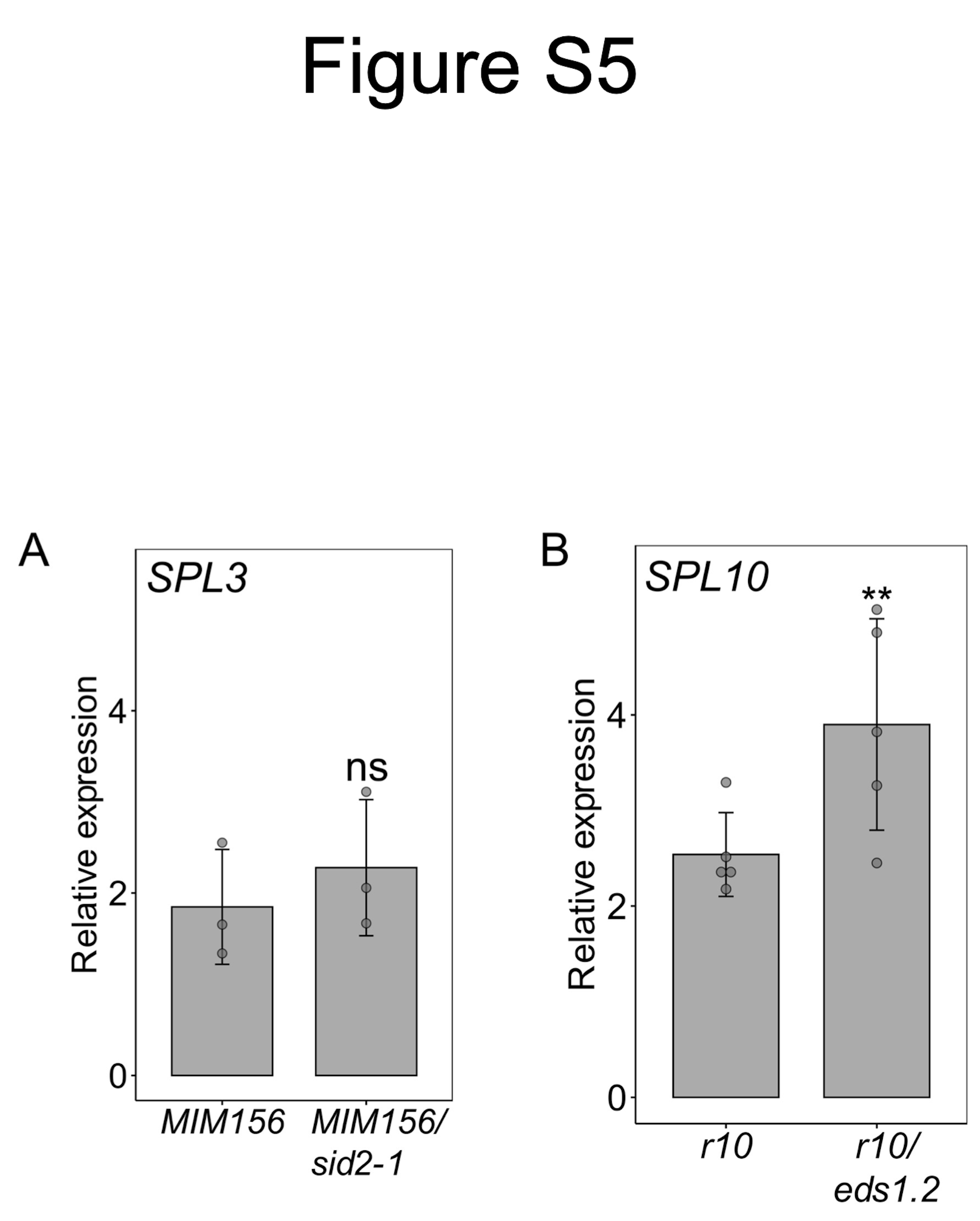

Supplement: S5 Fig — A, SPL3 was used as an indicator of MIM156 function. No difference was observed in MIM156 and MIM156/sid2-1. B, SPL10 was expressed at comparable level in rSPL10 and rSPL10/eds1.2. Student t test, ns, not significant, *, p < 0.05, **, p < 0.01. (TIFF) [file ppat.1011218.s005.tiff]

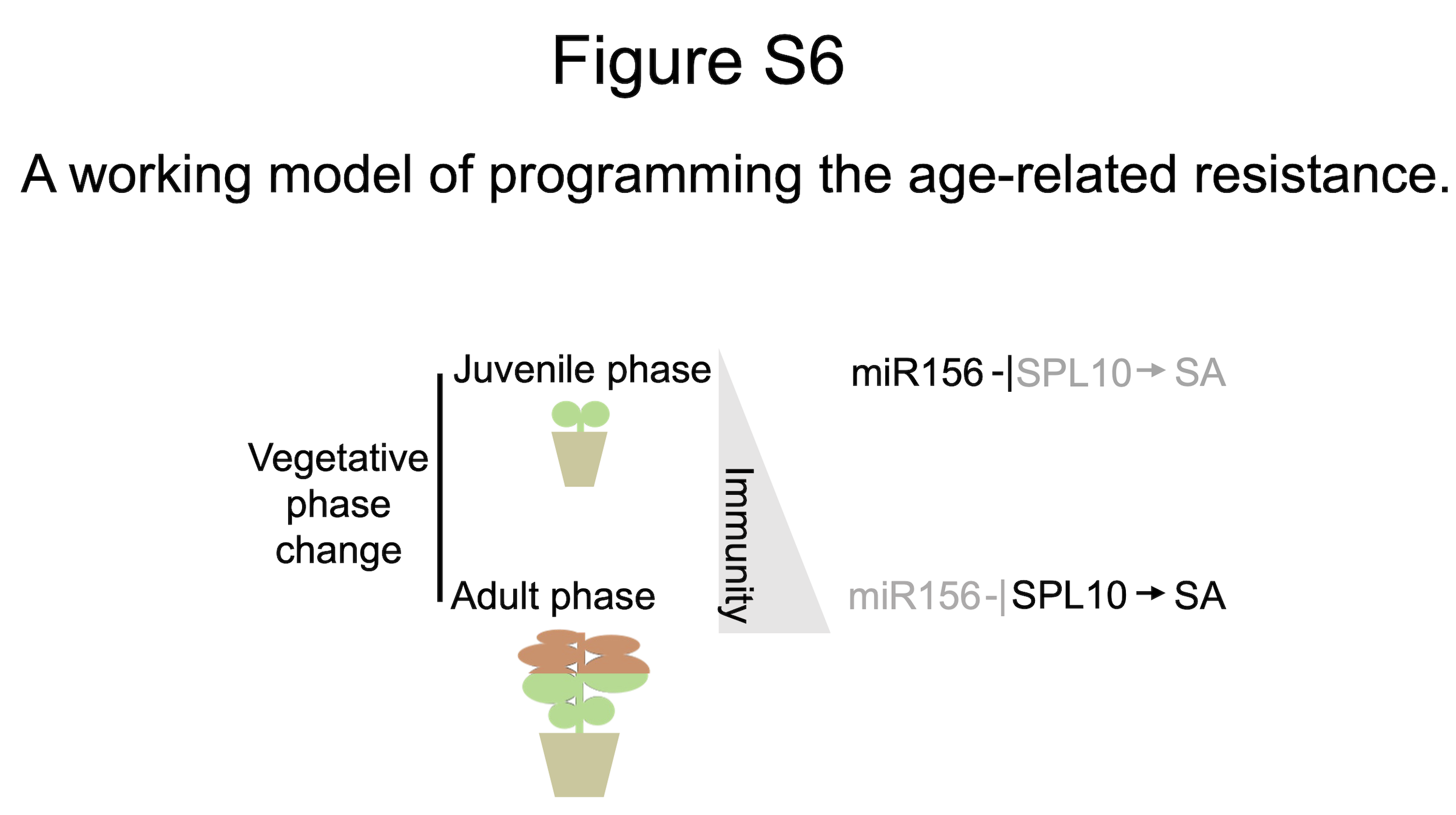

Supplement: S6 Fig — In brief, miR156 suppressed the resistance in juvenile phase through inhibiting SPL10. The increased expression of SPL10 followed by the decline of miR156 level gives rise to a high immune output in adult phase. That is achieved via promoting the expression of PAD4 as well as enhancing expressions of other components in SA biosynthesis and signaling pathways. (TIFF) [file ppat.1011218.s006.tiff]
